# The Use of MRI and TMS in Treatment-Resistant Depression: Advances in Pediatric Applications

**DOI:** 10.3390/brainsci15020194

**Published:** 2025-02-14

**Authors:** Trinh Ha, Katarina Jakimier, Sean O’Sullivan

**Affiliations:** Department of Psychiatry and Behavioral Sciences, Dell School of Medicine, Austin, TX 78712, USA; katarina.jakimier@utexas.edu (K.J.); sean.osullivan@utexas.edu (S.O.)

**Keywords:** pediatrics, depression, treatment-resistant, functional magnetic reasoning, major depressive disorder, transcranial magnetic stimulation

## Abstract

Treatment-resistant depression (TRD) is a substantial burden for psychiatric care, affecting approximately one-third of patients with major depressive disorder (MDD). Adolescent populations with depression are a particularly challenging demographic to treat as early intervention is crucial to prevent treatment resistance, but treatment options are limited. Transcranial magnetic stimulation (TMS) has emerged as a promising non-invasive option for TRD in adults as well as adolescents, offering hope for patients who have not responded to conventional therapies. This review examines the convergence of functional magnetic resonance imaging (fMRI) as a tool to examine how TMS modulates functional connectivity in adolescents with MDD. Such analyses have led to advances in our understanding of the pathophysiology of MDD, TRD, and the mechanisms of TMS. We review this evidence, evaluate methodological approaches, and identify critical gaps in the existing literature, highlighting how neuroimaging-guided TMS protocols offer a promising therapeutic avenue for adolescent TRD, particularly in cases where conventional treatments have proven ineffective.

## 1. Introduction

Pediatric depression is a multifaceted disorder with remarkable prevalence in both childhood and adolescence. Major depressive disorder (MDD) affects approximately 2.8% of children under the age of 13, with rates increasing to 5.6% during adolescence [1]. The etiology of pediatric MDD is complex; genetic predispositions, environmental stressors, and dysregulation of neurotransmitter systems have all been implicated in the development of depressive symptoms [2]. The course of pediatric depression is often protracted and recurrent, with early onset associated with a higher risk of persistent depressive episodes into adulthood [3]. Squires and colleagues [4] found that the rate of symptom improvement within the initial four weeks of treatment can effectively distinguish among pediatric patients who will achieve remission and those who will not.

Pediatric depression exerts striking and multifaceted impacts on a child’s life, affecting academic performance, social functioning, and neurodevelopment [5]. Children and adolescents with depression often exhibit marked declines in academic achievement, attributed to difficulties in concentration, memory, and motivation, which can negatively impact learning and result in long-term educational setbacks [6]. Socially, depressive symptoms contribute to withdrawal from peer interactions and decreased participation in activities, leading to impaired social skills and reduced support networks [7]. This social isolation can exacerbate feelings of loneliness and low self-esteem, perpetuating a cycle that further entrenches depressive states [8]. Neurobiologically, pediatric depression is associated with changes in brain structures critical for emotional and cognitive processing. Studies have shown that recurrent depressive episodes can alter the volume and function of regions such as the prefrontal cortex and the hippocampus, affecting executive functions and emotional regulation [2,9].

## 2. Treatment-Resistant Depression in Pediatric Populations

Treatment-resistant depression (TRD) refers to depressive disorders that do not respond to first-line treatments, including selective serotonin reuptake inhibitors (SSRIs) and cognitive behavioral therapy (CBT). McIntyre and colleagues [10] indicate that approximately 30% of children and adolescents diagnosed with depression will develop a treatment-resistant form, leading to prolonged functional impairment and reduced quality of life. TRD is frequently associated with more severe clinical presentations, higher rates of hospitalization, and a notably increased risk of suicide [10,11].

The onset of depression during adolescence represents a difficult clinical challenge, characterized by high rates of treatment resistance and poor response to first-line antidepressant interventions [12]. Recent data show that over four million American adolescents between the ages of 12 and 17 experienced a major depressive episode in 2020 [13]. Early onset, especially before adolescence, is associated with a higher risk of developing TRD due to the longer course of illness. Children and adolescents with early-onset depression are often exposed to prolonged periods of untreated or partially treated depression—which can result in neurobiological changes, such as alterations in the hypothalamic–pituitary–adrenal (HPA) axis, making subsequent treatments less effective [14].

Hospitalization during early depressive episodes is another significant risk factor for TRD [15]. These children frequently present with suicidal ideation, psychotic features, or a history of treatment failure, indicating a more resistant form of depression. Early hospitalization often correlates with a longer illness course and recurrent depressive episodes [16], contributing to poor long-term outcomes.

## 3. Neuroimaging Findings in Pediatric TRD

Magnetic resonance imaging (MRI) aids in revealing the pathological genesis of psychiatric disorders [17]. Brain imaging studies have revealed dysfunction in key neural structures—including the limbic, striatal, thalamic forebrain, and medial prefrontal cortex—that affect information processing, emotion regulation, and attentional control [18].

MDD involves dysregulation across multiple neural networks, particularly the dorsolateral prefrontal cortex, amygdala, dorsomedial frontal cortex, and anterior cingulate cortex [19]. Studies have documented hyperactivity in these regions, especially in the default mode network (DMN), which peaks during mental rest and may contribute to rumination [20]. Similarly, dysfunction in the salience network (SN), which directs attention to environmental stimuli, may explain the characteristic pessimistic perception in MDD patients [21].

The affective network regulates basic functions such as sleep, sexual drive, and eating [19]. Research consistently shows that individuals with MDD demonstrate both diminished gray matter volume and heightened, persistent emotional responses [22]. The cognitive control network (CCN), located in the frontoparietal circuit, mediates higher-order cognitive processes, including goal-directed decision-making, cognitive task-switching, and attention-dependent executive control mechanisms [22].

Structural magnetic resonance imaging (sMRI) has revealed significant alterations in brain morphology associated with pediatric TRD [23]. Multiple studies have documented reduced gray matter volume in the dorsolateral prefrontal cortex (DLPFC), particularly in the right hemisphere, with these changes strongly correlating with depression severity and treatment resistance [9]. The hippocampus shows bilateral volume reductions of 8–10%, with more pronounced changes observed in early-onset cases [2].

White matter alterations have also been identified as a significant feature of pediatric TRD, particularly in frontal–limbic pathways [24]. Studies have revealed reduced white matter integrity in the corpus callosum, specifically in the genu and splenium, suggesting disrupted interhemispheric communication [25].

The dorsolateral prefrontal cortex (DLPFC) has emerged as a crucial therapeutic target in treatment-resistant depression (TRD), supported by converging evidence from structural and functional neuroimaging studies [26]. The DLPFC functions as a critical hub for top-down emotional regulation and integrates cognitive and emotional processes. Its role in reward processing and motivated behavior makes it particularly relevant for treating depressive symptoms [26].

Recent research has highlighted two key findings regarding DLPFC function in TRD:

1. The relationship between DLPFC function and depression severity, where right DLPFC hyperactivity strongly correlates with symptom severity and volume reductions correlate with illness duration [9].
2. The importance of DLPFC connectivity patterns, particularly their anticorrelation with the subgenual anterior cingulate cortex (sgACC), which appears disrupted in TRD but may normalize with successful treatment [27].

These findings have direct clinical applications for transcranial magnetic stimulation (TMS) therapy, where functional connectivity patterns for optimal TMS targeting have emerged as a promising approach [28]. This neuroimaging evidence provides a strong foundation for understanding the neural basis of pediatric TRD and supports the rationale for targeted interventions. The convergence of structural and functional alterations in the DLPFC not only offers clear directions for therapeutic approaches, but also suggests that patient-specific connectivity patterns may inform more effective, personalized stimulation protocols in treatment planning [22].

## 4. Transcranial Magnetic Stimulation in Pediatric TRD

### 4.1. Conventional TMS Approaches

Transcranial magnetic stimulation (TMS) is a non-invasive neuromodulation technique that delivers targeted magnetic pulses through the scalp. When these magnetic fields penetrate the skull, they convert into electrical currents within the brain tissue, triggering neuronal depolarization in specific brain regions [29]. Single-pulse TMS creates brief neural responses primarily used for diagnostic purposes and brain mapping, while repetitive TMS (rTMS) induces lasting changes in neural activity through mechanisms similar to natural brain plasticity [30].

When delivered repeatedly, these pulses create lasting changes that persist beyond the stimulation period [31]. The effects depend on stimulation parameters: low-frequency stimulation (≤1 Hz) decreases cortical excitability through long-term depression, while high-frequency stimulation (≥5 Hz) increases it through long-term potentiation. Individual responses to these modulations can vary [31].

### 4.2. Comparative Analysis of TMS Protocols

The therapeutic application of rTMS in depression has focused particularly on the DLPFC [32]. This targeting strategy is supported by positron emission tomography (PET) studies showing reduced prefrontal glucose metabolism in depression, which normalizes with successful antidepressant treatment [33].

The evolution of TMS protocols in pediatric TRD demonstrates significant advancement in treatment approaches, each with distinct advantages and limitations. Conventional rTMS, utilizing 10 Hz stimulation in 37 min sessions delivered five times weekly for six weeks, has established a robust safety profile in adolescent populations [34]. This protocol delivers 3000 pulses per session, accumulating 18,000 pulses weekly while targeting the left DLPFC [35]. Despite its well-documented safety and efficacy, this conventional protocol has the drawback of an extended treatment duration requiring daily visits to the clinic for 4–6 weeks. The development of intermittent theta-burst stimulation (iTBS), a protocol that employs three 50 Hz bursts in 5 Hz intervals has not only increased the clinical convenience of TMS by delivering more pulses in less time (only 3 min for 600 pulses), but also the safety profile of TMS as it relates to seizures [36,37]. Moreover, the short duration of iTBS protocols and advancements in TMS stimulators have led to the development of accelerated iTBS, more than one treatment per day, which may have improved efficacy and symptom relief onset compared to conventional protocols [38,39,40,41].

One of these aiTBS protocols, Stanford Accelerated Intelligent Neuromodulation Therapy (SAINT), leverages a spaced-learning approach of 1 h in between 10 TMS sessions. This high-dose protocol delivers 1800 pulses per session, totaling 90,000 pulses across a 5-day treatment course. Protocols for rTMS typically involve one session per day, five days a week, for four to six weeks, with each session lasting approximately 30–40 min [9]. The SAINT TMS protocol condenses the treatment schedule: instead of daily sessions over several weeks, SAINT delivers multiple shorter sessions per day over just five days, significantly reducing the total duration of treatment while maintaining high efficacy [39]. SAINT’s accelerated protocol uses iTBS, a patterned form of TMS designed to mimic natural brain rhythms [42]. Importantly, SAINT incorporates individualized fMRI-guided targeting of the left DLPFC using a proprietary Food and Drug Administration (FDA) cleared algorithm of branching logic that precisely locates the subregion within the left dorsolateral prefrontal cortex (L-DLPFC) most anticorrelated to the sgACC.

## 5. Stanford Accelerated Intelligent Neuromodulation Therapy (SAINT) Protocol

SAINT is a novel, optimized approach to TMS for TRD. Its high response and remission rates are likely the result of a combination of variables including the total number of pulses, high rate of pulses, spaced-learning approach that considers axonal physiology optimizing synaptic plasticity, and precision targeting. Indeed, by targeting the subregion within the L-DLPFC most anticorrelated to the sgACC, SAINT likely mimics the antidepressant effects of sgACC deep brain stimulation [31].

The success of SAINT in TRD in adults raises questions regarding its efficacy in the pediatric population. In March 2024, the FDA cleared the first TMS stimulator for conventional rTMS as an adjunctive treatment for adolescents aged 15–21 with MDD [9,42]. This breakthrough has spurred research into iTBS, aiTBS, and protocols using precision targeting such as SAINT in adolescent depression [43,44,45].

### 5.1. Other Emerging TMS Protocols

Alternative TMS protocols continue to evolve, with several emerging approaches showing promise [46]. TBS has demonstrated particular advantages over conventional rTMS, notably in treatment efficiency [47]. TBS sessions commonly last only 3–10 min, compared with conventional rTMS sessions, which can last up to 40 min. Several different TBS clinical protocols have shown efficacy for treating MDD: iTBS applied to the left DLPFC; continuous TBS (cTBS) applied to the right DLPFC; and consecutive bilateral cTBS/iTBS applied sequentially to the right and left DLPFC, respectively, in the same session [46,47].

### 5.2. Integration of MRI and TMS 

The neural mechanisms of TMS remain incompletely understood, despite investigation with multiple neuroimaging techniques [48]. Although most studies utilize concurrent TMS and electroencephalogram tests (TMS-EEG), fMRI’s spatial resolution makes it particularly valuable for understanding functional connectivity. Traditional methods of combining TMS and fMRI faced significant technical challenges due to fMRI’s requirement for a homogeneous magnetic field.

The development of MR-compatible TMS systems has overcome traditional limitations by enabling simultaneous (’online’) TMS-fMRI experiments. These systems position the TMS stimulator outside the MR room and connect it to the TMS coil inside the scanner bore via a long cable that runs through the wall [49]. The selection of the MRI receive-coil is crucial for TMS-fMRI studies, as it determines both the accessibility of brain regions for TMS stimulation and the quality of fMRI signal detection. This setup allows for direct setup and measurement of the immediate effects of TMS on brain activity during fMRI scanning [49].

## 6. Optimization of Treatment Parameters

Understanding and optimizing treatment parameters represents a critical frontier in addressing pediatric depression, particularly in cases of TRD [3]. The personalization of depression treatment has evolved beyond traditional trial-and-error approaches, embracing sophisticated diagnostic tools and targeted interventions. At the genetic level, pharmacogenetic testing provides crucial insights into drug metabolism variations, enabling clinicians to select antidepressants that maximize therapeutic benefit while minimizing adverse effects in pediatric patients [50]. This approach proves particularly valuable in addressing the challenging cycle of medication trials often experienced by young patients with TRD. For instance, a clinician might use pharmacogenetic data to identify that a patient metabolizes certain SSRIs more rapidly, necessitating adjusted dosing or alternative medication choices.

Neuroimaging advances have improved neuronavigated rTMS targeting [51]. These imaging approaches allow clinicians to precisely map neural circuits and customize stimulation protocols based on individual brain architecture [8]. The integration of artificial intelligence has further enhanced this precision, with AI-driven tools analyzing complex symptom patterns and predicting treatment responses. This technological advancement enables clinicians to fine-tune the timing and intensity of various interventions, from CBT to combined treatment modalities, ensuring alignment with each child’s developmental stage and specific needs [52].

### Biomarker Development and Clinical Integration

The advancement of biomarker development in pediatric TRD encompasses multiple domains crucial for treatment optimization. Neuroimaging biomarkers have emerged as particularly valuable indicators, including structural markers such as DLPFC gray matter volume, hippocampal volume, and white matter integrity in the corpus callosum [53,54]. Functional connectivity measures, notably DLPFC-sgACC anticorrelation and DMN activity, provide crucial insights into treatment response prediction [55]. Neurophysiological biomarkers offer complementary information through EEG measures, including theta-gamma coupling and alpha asymmetry, alongside TMS-evoked potentials such as motor evoked potential amplitude and cortical silent period [56]. The analysis of molecular biomarkers, specifically inflammatory markers (interleukin-6, tumor necrosis factor-alpha, and C-reactive protein) and neuroendocrine indicators (cortisol rhythm and brain-derived neurotrophic factor), provides deeper insights into patient depression status [57]. By combining these different types of biomarkers, clinicians can better select appropriate patients for specific treatments and monitor their progress more effectively, which may lead to improved treatment outcomes and more efficient use of medical resources.

The optimization of TMS protocols for pediatric populations requires particular attention to developmental variability. Research demonstrates that protocol customization, especially when targeting the dorsolateral prefrontal cortex (DLPFC), must account for age-specific variations in brain maturation [58]. Young adolescents (around age 14) experience distinct neurobiological processes compared to their older counterparts (around age 19), characterized by ongoing grey matter reduction and synaptic pruning [59]. These younger patients typically exhibit greater variability in cortical excitability, requiring carefully calibrated, often lower stimulation thresholds to prevent overstimulation while supporting healthy neural development [60].

In contrast, older adolescents and young adults demonstrate more stable neural architecture, with increased white matter density and refined connectivity within prefrontal networks [61]. This maturation allows for more consistent and potentially more efficient TMS protocols. For example, while a 19-year-old might respond well to standard stimulation parameters, a 14-year-old may require protocols adjusted to support ongoing network stabilization, with parameters potentially requiring regular reassessment as development progresses [62].

The implementation of personalized treatment approaches, however, raises important ethical considerations regarding healthcare equity. The advanced diagnostic tools and specialized interventions central to personalized medicine often come with significant cost implications, potentially creating or exacerbating disparities in care access [63]. Healthcare systems must develop strategies to ensure that the benefits of personalized treatment reach all patient populations, not just those with the most resources. This might include developing tiered approaches to personalization, where some level of treatment customization remains accessible even when the most advanced diagnostic tools are not available.

Success in optimizing treatment parameters requires robust interdisciplinary collaboration among clinicians, researchers, and scientists. This collaboration ensures that personalization strategies continue to evolve based on emerging evidence while remaining practical for clinical implementation. Regular assessment of treatment outcomes, coupled with ongoing refinement of personalization approaches, helps maximize the impact of interventions while minimizing the risk of treatment failure in this vulnerable population [50].

Looking ahead, the field continues to develop more sophisticated approaches to treatment personalization. Adaptive protocols incorporating real-time feedback mechanisms show particular promise, allowing for dynamic adjustment of interventions based on patient response [64]. These emerging approaches, combined with our growing understanding of developmental neurobiology, offer hope for increasingly effective treatments for pediatric depression, particularly in challenging cases of TRD.

## 7. Discussion

### 7.1. Understanding Clinical Implications

The convergence of neuroimaging findings and therapeutic applications in pediatric treatment-resistant depression represents a significant advance in our understanding and treatment of this challenging condition. The identification of the DLPFC as a critical therapeutic target, supported by robust neuroimaging evidence, has particular significance for treatment optimization [27]. The demonstrated correlation between DLPFC function and treatment response provides a concrete example of how neuroimaging insights can directly inform therapeutic interventions.

### 7.2. Personalized Treatment Guidelines

Treatment personalization in pediatric TRD requires careful consideration of age-specific factors and clinical presentations [3]. Clinical factors significantly influence protocol selection, with symptom severity guiding the choice between standard and accelerated approaches. Comorbidity profiles necessitate specific modifications, such as adjusted pulse frequencies for anxiety disorders or enhanced targeting precision for attention-deficit/hyperactivity disorder (ADHD) [65]. Anatomical customization through individual fMRI-guided DLPFC localization and network connectivity-based adjustments ensures optimal treatment delivery [66]. Regular monitoring through weekly symptom assessments, cognitive function evaluation, and side effect tracking enables dynamic protocol adjustment, particularly when suboptimal responses or developmental transitions occur. A comprehensive approach to personalization, integrating developmental, clinical, and anatomical factors, could optimize TMS delivery for pediatric populations.

Additionally, it is important to note that TMS can be expensive. However, this treatment is intended for patients with TRD, a population that has exhausted multiple first-line and second-line interventions with minimal success. For these patients and their families, the financial burden of prolonged, ineffective treatments (multiple medication trials, psychotherapy, and hospitalization) can be substantial [67], and families may be more willing to pursue costlier treatment options. The high remission rates associated with TMS [42] offer a promising path that may, in the long run, reduce overall future healthcare costs typical of patients with TRD [8].

Compliance concerns are valid for any treatment in pediatric and adolescent populations; however, TMS presents unique advantages over traditional pharmacological treatments. Daily medication regimens require strict adherence and often result in missed doses or premature discontinuation due to side effects [5]. TMS is administered in a structured clinical setting under the guidance and supervision of professionals, reducing the likelihood of noncompliance [52]. Clinicians take active steps to ensure patient comfort throughout the treatment process to increase and maintain compliance, including ensuring a calm, therapeutic environment, providing earplugs, and seating patients in a comfortable, reclined position during TMS sessions. Additionally, the overall duration of TMS treatment is significantly shorter than the typical duration of medication regimens; this lowers the risk of noncompliance. Importantly, given that TMS is usually considered only after multiple failed treatments, patients undergoing this therapy are likely to be motivated to comply, as both they and their families recognize the need for a novel intervention.

### 7.3. Treatment Integration 

Personalizing TMS protocols for pediatric populations involves accounting for substantial individual variation in brain development and structural maturation. Protocol customization, particularly when targeting the DLPFC, can greatly enhance treatment outcomes by aligning stimulation parameters with each patient’s unique neural architecture [68,69].

### 7.4. Ethical Considerations

Healthcare disparities represent a fundamental ethical challenge in pediatric depression treatment, particularly with advanced interventions like TMS. Children from underserved communities face compounded barriers: not only must they overcome general obstacles to mental health care access, but TMS’s intensive nature—requiring multiple sessions over several weeks—creates additional logistical and financial burdens. Factors such as geographic location, socioeconomic status, and insurance coverage often determine whether a child can access these potentially life-changing interventions, creating a cascade of inequities that can affect their entire developmental trajectory.

## 8. Conclusions

The integration of MRI and TMS represents a significant advance in treating pediatric treatment-resistant depression. Early-onset depression manifests with distinct neurobiological signatures, including reduced grey matter volume in the DLPFC and disrupted functional connectivity patterns [2,9]. Recent advances in MRI-guided TMS protocols, particularly the FDA-approved SAINT protocol, leverage these insights through connectivity-based targeting and personalized parameters, while reducing traditional treatment burden [39]. The clinical implications of this integrated approach are substantial. Neuroimaging biomarkers enable early identification of treatment-resistant cases, allowing for targeted interventions that may prevent the cascading effects of pediatric depression on academic, social, and emotional development [14,60]. Moreover, personalized TMS protocols offer an alternative to traditional pharmacological treatments, potentially avoiding the challenges of medication side effects and lengthy trial-and-error approaches.

Several critical research priorities must be addressed to advance the field. First, the development of standardized protocols for neuroimaging-guided treatments and their validation through large-scale clinical trials across diverse patient populations is essential. These efforts should be accompanied by comprehensive cost-effectiveness analyses comparing these approaches to conventional therapies. Second, biomarker development should focus on multiple domains: neuroimaging markers such as DLPFC connectivity patterns and grey matter volume, neurophysiological indicators including theta-gamma coupling and event-related potentials, and molecular biomarkers of inflammation and stress response. These multimodal biomarkers could enable more precise patient selection and treatment monitoring, potentially improving response rates and optimizing resource allocation.

Longitudinal investigation represents a third crucial research priority, particularly given the developmental significance of adolescence. Studies must track patients from early adolescence (12–14 years) through late adolescence (17–19 years) and into early adulthood (20–25 years) to understand how early intervention shapes both clinical outcomes and neurodevelopmental trajectories [70]. This age range spans critical periods of prefrontal cortex maturation and network refinement, making it particularly relevant for understanding treatment effects. As our understanding of pediatric TRD’s neurobiological underpinnings grows, addressing these research priorities will be crucial in establishing neuroimaging-guided TMS as a standard treatment option for this vulnerable population.

Treatment-resistant depression in pediatric populations presents unique therapeutic challenges that require innovative, evidence-based interventions. The integration of advanced neuroimaging with TMS protocols has demonstrated significant potential in both mechanistic understanding and therapeutic applications [9,42]. Emerging evidence from multimodal biomarker studies and longitudinal investigations continues to elucidate the neurodevelopmental implications of early intervention [14,60]. Given the substantial evidence supporting neuroimaging-guided TMS in pediatric populations, along with its favorable safety profile and rapid onset of action, this therapeutic approach merits consideration as a first-line treatment for adolescent treatment-resistant depression, particularly in cases where conventional interventions have proven ineffective.

## Data Availability

No new data were created for this review.

## Abbreviations

The following abbreviations are used in this manuscript:

ADHD	Attention Deficit Hyperactivity Disorder
CBT	Cognitive Behavioral Theory
CCN	Cognitive Control Network
DLPFC	Dorsolateral Prefrontal Cortex
DMN	Default Mode Network
EEG	Electroencephalogram
FDA	Food and Drug Administration
fMRI	Functional Magnetic Resonance Imaging
HPA	Hypothalamic–Pituitary–Adrenal
i-TBS	Intermittent Theta Burst Stimulation
L-DLPFC	Left Dorsolateral Prefrontal Cortex
MDD	Major Depressive Disorder
MR	Magnetic Resonance
MRI	Magnetic Resonance Imaging
PET	Positron Emission Tomography
rTMS	Repetitive Transcranial Magnetic Stimulation
SAINT	Stanford Accelerated Intelligent Neuromodulation Therapy
sgACC	Subgenual Anterior Cingulate Cortex
sMRI	Structural Magnetic Resonance Imaging
SN	Salience Network
SSRI	Selective Serotonin Reuptake Inhibitor
TBS	Theta Burst Stimulation
TMS	Transcranial Magnetic Stimulation
TRD	Treatment-Resistant Depression
